# The effectiveness of e-& mHealth interventions to promote physical activity and healthy diets in developing countries: A systematic review

**DOI:** 10.1186/s12966-016-0434-2

**Published:** 2016-10-10

**Authors:** Andre Matthias Müller, Stephanie Alley, Stephanie Schoeppe, Corneel Vandelanotte

**Affiliations:** 1 Centre for Community and Clinical Applications of Health Psychology; Faculty of Social, Human and Mathematical Sciences, University of Southampton, Southampton, SO17 1BJ UK; 2 Physical Activity Research Group, School of Human, Health and Social Sciences, Central Queensland University, Building 77, Bruce Highway, Rockhampton, QLD 4702 Australia

**Keywords:** Web-based, Mobile phone, ICT, Technology, Developing countries, Dietary transition, NCDs, Exercise, Healthy eating

## Abstract

**Background:**

Promoting physical activity and healthy eating is important to combat the unprecedented rise in NCDs in many developing countries. Using modern information-and communication technologies to deliver physical activity and diet interventions is particularly promising considering the increased proliferation of such technologies in many developing countries. The objective of this systematic review is to investigate the effectiveness of e-& mHealth interventions to promote physical activity and healthy diets in developing countries.

**Methods:**

Major databases and grey literature sources were searched to retrieve studies that quantitatively examined the effectiveness of e-& mHealth interventions on physical activity and diet outcomes in developing countries. Additional studies were retrieved through citation alerts and scientific social media allowing study inclusion until August 2016. The CONSORT checklist was used to assess the risk of bias of the included studies.

**Results:**

A total of 15 studies conducted in 13 developing countries in Europe, Africa, Latin-and South America and Asia were included in the review. The majority of studies enrolled adults who were healthy or at risk of diabetes or hypertension. The average intervention length was 6.4 months, and text messages and the Internet were the most frequently used intervention delivery channels. Risk of bias across the studies was moderate (55.7 % of the criteria fulfilled). Eleven studies reported significant positive effects of an e-& mHealth intervention on physical activity and/or diet behaviour. Respectively, 50 % and 70 % of the interventions were effective in promoting physical activity and healthy diets.

**Conclusions:**

The majority of studies demonstrated that e-& mHealth interventions were effective in promoting physical activity and healthy diets in developing countries. Future interventions should use more rigorous study designs, investigate the cost-effectiveness and reach of interventions, and focus on emerging technologies, such as smart phone apps and wearable activity trackers.

**Trial registration:**

The review protocol can be retrieved from the PROSPERO database (Registration ID: CRD42015029240).

**Electronic supplementary material:**

The online version of this article (doi:10.1186/s12966-016-0434-2) contains supplementary material, which is available to authorized users.

## Background

In 2012, about 38 million global deaths were attributed to non-communicable diseases (NCDs) such as diabetes, cardiovascular diseases and cancer, and it is expected that NCD death rates will increase further, reaching 52 million by 2030. The NCD burden is particularly high in developing countries with 82 % of global NCD-related deaths occurring in low and middle income countries [1, 2].

Major NCD prevention strategies include the reduction of behavioural risk factors, especially physical inactivity and unhealthy diets [1, 3–6]. There is extensive evidence on the preventive effects of regular physical activity and healthy eating on the risk of developing a NCD [7, 8]. For example, a 25 % reduction in physical inactivity is estimated to prevent about 1.3 million NCD-related deaths annually [9] while a healthy diet and increased physical activity can prevent a significant proportion of the 18 million deaths caused by high blood pressure, high body mass index, high fasting blood glucose and high total cholesterol [10].

In developing countries, rapid globalization is contributing to a change in people’s diets where local low calorie and high fibre foods are replaced by readily available, cheap and processed foods high in fat, salt and sugar [8, 11]. For example, in developing countries in Asia, the consumption of processed foods increased by more than 5 % between 1999 and 2012. In contrast, the consumption of processed foods in developed countries increased only by 0.2 % [12]. Additionally, rapid technological development decreases the necessity of physical labour and active transport which in turn contributes to decreasing levels of physical activity [8, 13, 14]. Decreasing physical activity levels were observed in most Asian [15, 16], Latin-and South American [17–19] and some African countries [13, 20] as urbanization increased.

One promising way to promote physical activity and healthy diets in developing countries is to implement electronic and mobile health (e-& mHealth) interventions. These interventions are primarily delivered via modern information and communication technologies (ICT) such as the Internet, mobile phones, and other wireless devices [21]. The proliferation of such ICTs is very high in developing countries. For example, in 2015, 90 % of people living in developing countries owned a mobile phone and two thirds of the global Internet users were based in developing countries [22, 23]. Therefore, it is feasible, and potentially cost-effective, to reach large numbers of people using ICTs in developing countries.

Currently, the evidence on physical activity and behavioural diet e-& mHealth interventions is largely drawn from reviews that did not include studies conducted in developing countries [21, 24, 25]. For example, a recent review on mHealth for the prevention of cardiovascular diseases, only retrieved studies conducted in developed countries [24]. The same applies to reviews focusing on Internet [25, 26], social media [27], smart phone [28, 29], and mobile phone text messaging [30, 31] interventions to promote physical activity and healthy diets. Furthermore, a recent review focussing on mHealth interventions in patients with an NCD from developing countries identified only two studies that measured physical activity and none examined dietary behaviours [32]. A systematic review of the research literature on physical activity and diet e-& mHealth interventions conducted in the developing world is currently lacking.

To address this gap, the objective of this systematic review was to investigate the effectiveness of e-& mHealth interventions to promote physical activity and healthy diets in developing countries.

## Methods

This review was conducted and is reported according to the PRISMA guidelines [33] and the protocol can be retrieved from the PROSPERO database (Registration ID: CRD42015029240).

### Study eligibility criteria

Studies were included if they a) quantitatively examined the effect of an e-& mHealth intervention on physical activity and/or diet outcomes; b) were conducted as a quasi-experimental trial, cross-over trial, controlled trial (CT), or randomized controlled trial (RCT); c) included participants from a developing country; and d) were published in English. Only studies in which the e-& mHealth component was the main or a major intervention delivery mode were included. E-& mHealth was defined as the use of ICT to promote physical activity and/or healthy diets. Interventions that were delivered via the Internet (webpages, social media, and email), mobile phone text messages or mobile phone calls, smartphone technology (‘apps’) and other wireless devices (e.g. wearable activity trackers, tablets) were included [21]. The current World Bank classification (July 2015) was used to determine developing country status (low income, lower-middle income, and upper-middle income) [34]. Studies were still included when they were simultaneously conducted in developing and developed countries [35–37]. Every search record was assessed against the inclusion criteria by one of the reviewers (AMM, SA, SS). In cases where study inclusion was unclear a decision was made via discussion including all reviewers.

The primary outcomes of this review were objectively or subjectively measured physical activity and/or dietary behaviour. This could be changes in physical activity levels, time spent doing physical activity, adherence to physical activity recommendations, energy expenditure, step counts, exercise/sport participation, active transport, sedentary time, accelerometer counts; food frequency, diet quality (as defined in the respective studies), fruit and vegetable intake, consumption of sweetened beverages and foods high in sugar, salt or saturated fat (or ultraprocessed foods), dairy product consumption, consumption of fat and dietary fibre, meal size, or caloric intake. Indirect calorimetry, body composition, BMI, body weight, waist circumference, waist-hip ratio, body fat, and lean body mass were reported if available, but only as secondary outcomes.

### Information sources and search

A systematic search was performed in the following databases: Cochrane Central Register of Controlled Trials (CENTRAL) and the Health Technology Assessment Database (HTA; Cochrane Library), EBSCOHOST (including SPORTDiscuss, CINAHL, MEDLINE, PsycARTICLES, Psychology and Behavioral Science Collection), SCOPUS, Web of Science Core Collection and the World Health Organization Global Health Library. For each database, search terms were combined with the appropriate Boolean Operators: technology or email or internet etc. AND physical activity or exercise or walking etc. or healthy eating or nutrition or sugar intake etc. AND developing country or low-income country etc. or Afghanistan or Albania etc. The search period covered the date range 2000 to 31st October 2015 where available in the database. The data base search (Additional file 1 Cochrane Library) was piloted by the corresponding author and reviewed by all co-authors.

Additionally, articles were hand-searched in the grey literature and reference lists of relevant papers were reviewed. Moreover, the Johns Hopkins Global mHealth Initiative was contacted and a request for relevant studies was posted on ResearchGate (social media network for scientists) and created article alerts to derive 2016 articles outside the systematic search to be as current as possible (last inclusion was made in August 2016).

### Data extraction

A data extraction form was developed based on previous reviews on physical activity and diet interventions [29, 30]. It was piloted on four studies by the corresponding author and refinements were made based on the feedback of the co-authors. Data on study setting/location and participants, e-& mHealth intervention characteristics and intervention effectiveness were retrieved. One reviewer extracted the relevant study information and a second reviewer assessed the data for accuracy and completeness. Disagreement between the reviewers was resolved through discussion and consensus with a third reviewer.

The risk of bias assessment was conducted independently by two reviewers using the CONSORT checklist (AMM, SA) [38] which has been used in previous e-& mHealth reviews [27]. This checklist consists of 25 criteria. If a study fulfilled a criterion it received one point. Studies received half a point if it fulfilled one of two points making up a criterion. A higher overall score indicated lower methodological bias. The obtained risk of bias score of each study was divided by 25 (highest attainable score) and multiplied by 100 to obtain the percentage of fulfilled criteria. Disagreement between the reviewers was resolved through discussion and consensus with a third reviewer (SS). Studies were then grouped into low (>66.7 % fulfilled criteria), moderate (50–66.7 % fulfilled criteria) and high risk of bias (<50 % fulfilled criteria) [25, 39].

If available, changes in physical activity and diet between baseline and intervention completion, and between baseline and final follow-up (period following an intervention) were presented. If possible, effect sizes were reported or calculated (e.g., Cohen’s *d*, mean difference of change between groups), and confidence intervals and significance levels were presented. Where possible the between group effects were presented. The significance level was set to *p* ≤ .05.

As few studies provided data needed to calculate effects sizes and due to the great variability in study designs, interventions, and outcome measures conducting a meta-analysis was not possible.

## Results

### Study selection

**Fig. 1:**
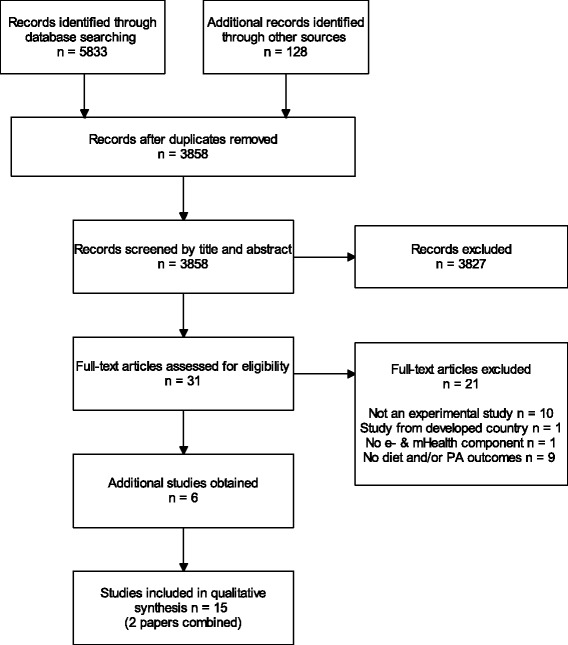
Flowchart for the selection of studies on e-& mHealth interventions to promote physical activity and healthy diets in developing countries

A total of 5961 publications including 2231 duplicates were identified through the date base search. After screening the titles and abstracts of 3858 publications, the full-text of 31 publications was assessed for study eligibility. Of these, 10 publications were included in the review. Another six publications were identified through citation alerts and reference list checks. Finally, two publications reported on the same study [40, 41]. As such, a total of 16 publications describing 15 distinct studies were included in the current systematic review (Fig. 1).

### Study characteristics

Table 1 provides an overview of the study characteristics. The majority were RCTs (*n* = 8) with two (*n* = 7) [36, 40, 42–46] or three group study designs (*n* = 1) [35]. The remaining seven studies were CTs (*n* = 3) [47–49] or quasi-experimental trials (*n* = 4) [37, 50–52]. The e-& mHealth interventions lasted between 1 and 24 months (median = 4 months, mean = 6.4 months). In eleven studies physical activity and/or diet outcomes were assessed only at baseline and immediately after the e-& mHealth intervention [35–37, 40, 42, 45, 47–49, 51, 52]. Three studies also conducted follow-up assessments after intervention conclusion (3-month follow-up for all 3 studies) [44, 46, 50] and in one study, physical activity and diet outcomes were assessed at baseline, during the intervention and at intervention completion [43].

**Table 1:** Characteristics of behavioural e-& mHealth intervention studies included in the review

Author Year Country	Study design Duration Sample (sample size, mean age/range, sex)	Intervention	Outcome measures (PA, SB, diet)	Retention rate Acceptability Participation rate	Results
Lana et al. 2014 [35] Mexico and Spain	Study design 3-group RCT Duration Intervention exposure: 9 months Measurement points: baseline and 9 months Sample *N* = 2001 (737 analysed); 12–16 years students; 45.2 % (M), 54.8 % (F)	IG 1 & IG 2: Website targeting cancer risk behaviours (advantages of healthy/disadvantages of risky behaviours, skills training to avoid risk behaviours, expert advice, videos, forums, documents, web links, educational games) based on the Theory of Planned Behavior and the Transtheoretical Model IG 2: Additional weekly text messages encouraging health behaviours CG: No intervention	Behavioural outcomes Diet (fruit and vegetable intake, fat intake); PA (doing PA less than 360 min/week) Measures Online questionnaire Other relevant outcomes BMI	Retention 36.8 %	Diet: Sig. within-group increase in percentage of students consuming enough fruits in all groups (67.0 % mean decrease, *p* < .001); no sig. within-group changes for other diet behaviours PA: No sig. within-groups change in percentage of students doing less than 360 min/week PA BMI: Sig. within-group changes in percentage of overweight/obese students in IG 2 (19.6 % decrease, *p* < .05)
Rotheram–Borus et al. 2012 [50] South Africa	Study design 1-group pre-post-follow-up Duration Intervention exposure: 3 months; Measurement points: baseline, 3 and 6 months *N* = 22; 53.0 year/21–74 years diabetic township residents; 100 % (F)	3-component PA and diet program: Weekly educational group sessions addressing healthy lifestyle; daily text messages asking about adherence to healthy behaviours; peer support for lifestyle changes via text messages or call	Behavioural outcomes PA (daily step count) Measurements Pedometer Other relevant outcomes BMI	Retention 100 % Participation rate Participants responded to 54 % of text messages sent by study team; peers exchanged on average 123 text messages weekly	PA: No sig. change in daily step counts from baseline to 3 months (*d* = 0.03), and from baseline to 6 months (*d* = 0.27) BMI: No sig. changes (*d* = −0.07 to 0.07)
Ramachandran et al. 2013; Ram et al. 2014 [40, 41] India	Study design Prospective 2-group RCT Duration Mean duration of follow-up 20.2 months (SD 7.0), either intervention exposure of 24 months or until participants developed diabetes Sample *N* = 537 (517 analysed); 46.0 year/35–55 years working men with impaired glucose tolerance; 100 % (M)	IG & CG: Face-to-face education and motivation about healthy lifestyle plus written information about diet and PA (balance food intake and PA to achieve/maintain healthy body weight IG: Additional 2–4 weekly text messages; messages based on the Transtheoretical Model and contained information about diet and PA, benefits of healthy diet and PA, strategies for relapse prevention and motivation to maintain healthy diet and PA	Behavioural outcomes Diet (total dietary energy intake, adherence to dietary recommendations, portion size, oil intake, carbohydrate consumption); PA (PA score ranging from 7 to 70, adherence to PA recommendations) Measurements Diet (24 h recall), PA (own questionnaire) Other relevant outcomes BMI, waist circumference	Retention 96 % Acceptability Test messages were welcomed, 3 % were disturbed by text messages at least once	Diet: Sig. difference in mean change = −43.7 kcal/day (95 % CI:−65.5;−22.0) favouring IG; Sig. more participants in IG adhered to dietary recommendations at follow-up (OR 1.36, 95 % CI: 1.01; 1.83); higher percentage of participants in IG improved portion size OR = 0.39 (95 % CI: 0.25; 0.60), oil intake OR = 0.46 (95 % CI: 0.30; 0.69), carbohydrate consumption OR = 0.52 (95 % CI: 0.34; 0.78) vs CG (*p* < .05) PA: Non-sig. difference in mean change in PA score = −1.0 point (95 % CI:−2.0; 0); Adherence to PA recommendations did not sig. differ at follow-up (OR 1.11, 95 % CI: 0.78; 1.57, *p* > .05) BMI: Difference in mean change = −0.05 kg/m^2^ (95 % CI: −0.46; 0.37) Waist circumference: Difference in mean change = 0.04 cm (95 % CI: −0.56; 0.64) The mean lifestyle score was higher in the IG than the CG (2.59 ± 1.13 vs. 2.28 ± 1.17; *p* = .002)
Shetty et al. 2011 [42] India	Study design 2-group RCT Duration Intervention exposure: 12 months Measurement points: baseline and 12 months Sample *N* = 215 (144 analyzed); 50.3 years/type 2 diabetic patients; Both sexes (no further information)	IG & CG: During initial and follow-up visits education program with individual advice on nutrition and PA IG: Additional 2–4 weekly text messages; reminders/instructions to follow regimen of healthy diet and PA; messages on healthy habits	Behavioural outcomes Diet (scores for components of healthy diet and frequency of adherence to it); PA (scores for occupation and leisure time PA) Measurements Questionnaire Other relevant outcomes BMI	Retention 67 % Acceptability Text messages highly acceptable reminder tool	Diet: No sig. changes in percentage of participants adhering to diet regiment (IG: from 60.3 to 58.4 %; CG: from 54.5 to 52 %) PA: No sig. changes in percentage of participants complying with PA advice (IG: 47 to 56 %; CG: 47 to 52 %) BMI: No sig. changes
Zolfaghari et al. 2012 [47] Iran	Study design 2-group CT Duration Intervention exposure: 3 months Measurement points: baseline and 3 months Sample *N* = 80 (77 analysed); 18–65 years diabetes patients; 53 % (F) 47 % (M)	IG1: Phone counselling about diabetes management including health behaviour twice weekly for 1st month and weekly for months 2 and 3. IG2: 6 weekly text messages on diabetes management including behavioural health.	Behavioural outcomes Adherence to diet and PA recommendations as a score Measurements Questionnaire	Retention 96.3 %	Diet Adherence: Sig. within-group increase in IG1 (18.24 ± 2.46, *p* < .001) and IG2 (16.50 ± 1.98, *p* < .001) but no sig. between-group changes (*d* = −0.78, *p* = .44) PA Adherence: Sig. within-group increase in IG1 (35.66 ± 0.68, *p* < .001) and IG2 (40.02 ± 1.43, *p* < .001) but no sig. between-group changes (*d* = 4.13, *p* = .33)
Chen et al. 2014 [51] China	Study design 1-group pre-post Duration Intervention exposure: 1 to 6 months Measurement points: baseline and 1 to 6 months Sample *N* = 253; 40+ yrs pre diabetic patients in rural area; 68 % (F) 32 % (M)	Computer tailored web-based intervention for diabetes prevention. Delivered each time a patient presents at medical clinic to see general practitioner. Includes education, diabetes risk scoring and tailored feedback on changes on lifestyle behaviours (diet and PA) and barriers. Prompts general practitioner.	Behavioural outcomes PA (one question at follow up to determine increased leisure time exercise) Diet (two questions at follow up to determine reduced calorie intake and increased fruit and vegetable intake) Measurements Interview Other relevant outcomes Body weight, BMI	Retention 91 % Acceptability 8.76–9.20 out of 10.	PA: Sig. change in number of participants who increased leisure time exercise from 16 (6.3 %) to 49 (21.2 %, *p* < .001) Diet: Sig. increase in number participants who reduced caloric intake from 4 (1.6 %) to 165 (71.4 %, *p* < .001); Sig. increase in number of participants who increased fruit-and vegetable intake from 43 (17 %) to 205 (88.7 %, *p* < .001) BMI: Sig. reduction from 24.8 kg/m^2^ (±3.21) to 23.4 kg/m^2^ (±2.95) (*d* = 0.49, *p* < .001) Body weight: Sig. reduction from 62.1 kg (±9.85) to 58.3 kg (±9.18) (*d* = 3.43, *p* < .001)
Tamban et al. 2013 [43] Philippines	Study design 2-group RCT Duration Intervention exposure: 6 months Measurement points: baseline, 3 months and 6 months Sample *N* = 125 (104 analysed); 19–50 year diabetes patients; 48 % (F) 52 % (M).	IG & CG: Lecture from diabetes educator and usual appointments with diabetes educator and endocrinologist. IG: Additional text messages 3 times weekly for 6 months on healthy diet, exercise and consequences of negative health behaviours.	Behavioural outcomes PA (adherence to 30 mins of exercise on 5 days weekly) Diet (Number of meals meeting diet recommendations and number of days adhered to 3 proper meals recommendation) Measurement Interview Other relevant outcomes BMI, Body weight	Retention 79 %	PA: Sig. between-group increase in minutes of exercise at 6 months favouring the IG (*p* = .02); no sig. between-group changes in mean number of days meeting PA recommendations Diet: Sig. between-group improvements in adherence to 3 meals per day recommendation favouring IG (*p* = .02); no sig. between-group changes in mean number of days meeting diet recommendations. BMI: No sig. between-within group changes Body weight: No sig. between-within group changes
Nurgul et al. 2015 [52] Turkey	Study design 1-group pre-post Duration Intervention exposure: 3 months Measurement points: baseline and 3 months Sample *N* = 44 (30 analysed); 18–55 years university employees; 100 % (F)	Web-based health intervention: Modules delivered every 3 weeks. 1 module on nutrition, 1 on diet and 1 on smoking and stress. Modules consist of an audio-visual lecture.	Behavioural outcomes PA and Diet (Health Promotion Lifestyle Profile) Measurement Online Questionnaire	Retention 68.2 %	PA: Sig. increase from 16.63 points (±5.33) to 19.20 points (±5.25), *d* = −0.48, *p* = .004 Diet: Sig. increase from 20.70 points (±3.90) to 23.47 points (±3.41), *d* = −0.81, *p* = .001
Bombem et al. 2013 [48] Brazil	Study design 2-group CT Duration Intervention exposure: 6 months Measurement points: baseline and 6 months Sample *N* = 279 (236 analysed); 18–64 years adult employees; 42.3 % (M), 57.7 % (F)	IG: Healthy Weight Program incl. dietary and PA education through tailored monthly email messages, as well as goal setting, and self-monitoring of weight. Based on Social Cognitive Theory. CG: Wait-list control Healthy weight program at the end of 6-months intervention	Behavioural outcomes Diet (food and beverage intake incl. fruits, vegetables, grains, dairy, meat, legumes; fat and sodium intake) Measures 24 h dietary recall, phone interview	Retention 85 %	Diet: Sig. decrease in overall diet quality score in both groups (*p* < .05). Sig. more decrease in diet quality score in CG compared to IG (adjusted impact: 3.55, 95 % CI: 1.52; 5.57). Sig. increase in grains, but decrease in vegetable consumption, meat, eggs, sodium intake, and overall diet quality score (*p* < .05).
Sriramatr et al. 2014 [44] Thailand	Study design 2-group RCT Duration Intervention exposure: 3 months Measurement points: baseline, 3 and 6 months Sample *N* = 110; 19.0 year/18–24 years students; 100 % (F)	IG: Website and weekly emails incl. PA education, tailored advice, goal setting and self-monitoring via pedometer. Based on the Social Cognitive Theory. CG: Pedometer without website and emails.	Behavioural outcomes PA (daily step count, weekly leisure-time PA score) Measurements Online Questionnaire Pedometer	Retention 79 % Participation Rate 90–95 % accessed website, recorded PA and set PA goals each week	PA daily step counts: Mean difference in change from baseline to 3 months between groups was 3766 steps favouring IG. Mean difference in change from baseline to 6 months between groups was 3360 steps favouring IG PA leisure time activity score: Mean difference in change from baseline to 3 months between groups was 15.13 points favouring IG. Mean difference in change from baseline to 6 months between groups was 14.87 points favouring IG
Shahid et al. 2015 [45] Pakistan	Study design 2-group RCT Duration Intervention exposure: 4 months Measurement points: baseline and 4 months Sample *N* = 440; 49.08 years/18–70 year, type-2 diabetes patients; 61.4 % (M), 38.6 % (F)	IG & CG: Usual care plus leaflet on diet and a healthy lifestyle IG: Additional regular (every 15 days) mobile phone calls to provide feedback on self-monitored blood glucose levels over the past readings of 15 days.	Behavioural outcomes Diet; PA (if they are following diet plan and are physically active) Measures Not reported Other relevant outcomes BMI	Retention Not reported	Diet: Sig. increase in proportion of participants following dietary plan from baseline (17.3 %) to 4 months (43.6 %) in IG (*p* < .001). Non-sig. in CG PA: Sig. increase in proportion of physically active participants from baseline (16.4 %) to 4 months (44.5 %) in IG (*p* < .001) Non-sig. in CG BMI: Sig. reduction (*p* < .001) in IG (.96 ± .09) and CG (1.02 ± .09); *d* of difference in change − 0.67 favouring CG
Müller et al. 2016 [46] Malaysia	Study design 2-group RCT Duration Intervention exposure (text messaging): 3 months Measurement points: baseline, 3 and 6 months Sample *N* = 43 (39 analysed); 63.3 years/55–70 year, 26 % (M), 74 % (F)	IG & CG: Printed exercise booklet with 12 age appropriate exercises. IG: Additional 60 encouraging text messages over 3 months (content based on effective Behavior Change Techniques	Behavioural outcomes PA (weekly exercise frequency using the exercise booklet; PA-related energy expenditure; daily time spent sitting) Measures Exercise diary International PA Questionnaire (short) Other relevant outcomes BMI	Retention 86 % Acceptability IG participants liked the text messages and those who faced exercise barriers benefited from them.	PA (exercise frequency): Sig. more often exercise in IG (3.7 ± 1.3) compared to CG (2.5 ± 1.85) at 3 months (*d* = 0.76, *p* = .027); Non-sig. difference at 6 months (3.1 ± 1.3 vs. 2.3 ± 1.9, *d* = 0.45, *p* = .18) PA (PA related energy expenditure): No sig. between-within group changes PA (daily time spent sitting): No sig. between-within group changes BMI: No sig. between-group changes
Rubinstein et al. 2016 [36] Peru, Argentina, Guatemala	Study design 2-group RCT Duration Intervention exposure: 12 months Measurement points: baseline and 12 months Sample *N* = 637 (553 analysed); 43.4 years/30–60 year, adults with prehypertension 46 % (M), 54 % (F)	IG and CG: Leaflet with information on adoption of healthy lifestyle IG: Additional monthly calls to motivate participants to adhere to healthy behaviours (diet and PA) plus max. 5 text messages per month that were based on the Transtheoretical Model (target on chosen diet/PA behaviour).	Behavioural outcomes PA (weekly MET-minutes) Diet (daily intake of sodium, fat and sugar, fruits and vegetables) Measures International PA Questionnaire (short) Food Frequency Questionnaire Other relevant outcomes BMI, body weight, waist circumference	Retention: 86.8 % Acceptability: Participants found call and text messages helpful Participation rate: Only 3 % received all 12 calls, call duration 20–30 min, median of 23 text messages over 12 months	PA: Mean difference in change between groups − 80.4 (95 % CI:−386; 225.5, *p* = .61) Diet (daily sodium intake): Mean difference in change between groups − 0.07 (95 % CI:−0.25;0.12 *p* = .49) Diet (daily fat and sugar intake): Mean difference in change between groups − 0.75 (95 % CI:−1.30;−0.20, *p* = .008) Diet (daily intake of fruits and vegetables): Mean difference in change between groups 0.25 (95 % CI:−0.01; 51, *p* = .05) BMI: Mean difference in change between groups − 0.30 (95 % CI: −0.59; 0.06, *p* = .02) Body weight: Mean difference in change between groups −0.66 (95 % CI: −1.24; −0.07, *p* = .04) Waist circumference: Mean difference in change between groups −0.64 (95 % CI: −1.62; 0.35, *p* = .21)
Ganesan et al., 2016 [37] 92 % of participants from developing countries (India, China, Philippines)	Study design 1-group pre-post Duration Intervention exposure: 2.5 months Measurement points: baseline and 2.5 months Sample *N* = 69219 (36652 analysed); 36.0 year (±9 years), adult employees; 76.1 % (M), 23.9 % (F)	100-day Stepathlon programme: Participants received pedometer and entered daily step count into Stepathlon website or app. Website to facilitate motivation and engagement via self-monitoring, social networking, quizzes, expert chats and competition between employees. Encouraging emails daily and when milestones were reached.	Behavioural outcomes PA (daily step count, weekly exercise days, daily sitting time) Measures Pedometer Online survey Other relevant outcomes Body weight	Retention: 53.0 %	PA (daily step count): Sig. increase of 3519 steps (95 % CI: 3484; 3553, *p* < .001) PA (weekly exercise days): Sig. increase of 0.89 days/week (95 % CI: 0.87; 0.92, *p* < .001) PA (daily sitting time): Sig. decrease of 0.74 h/day steps (95 % CI:−0.78;−0.71, *p* < .001) Body weight: Sig. reducion of 1.45 kg (95 % CI:−1.53;−1.38, *p* < .001)
Pfammatter et al., 2016 [49] India	Study design 2-group CT Duration Intervention exposure: 6 months Measurement points: baseline and 6 months Sample *N* = 1925 (1243 analysed); 32.2 years (±10.6 years); 88.52 % (M), 11.48 % (F)	IG: 56 motivational text messages addressing awareness of diabetes and diabetes risk behaviours CG: No intervention	Behavioural outcomes PA (current exercise) Diet (fruit, vegetable and fat intake) Measures Telephone survey	Retention: 64.6 %	PA: No sig. between-group change in exercise participation (*p* > .05) Diet (daily intake of fruit and vegetables): Sig. between-group increase favouring the IG (*p* < .001) Diet (fat intake): Sig. between-group decrease favouring the IG (*p* < .001)

Abbreviations: *IG* intervention group, *CG* control group, *PA* physical activity, *BMI* body mass index in kg/m^2^, *MET* metabolic equivalent of task

One study was conducted in Europe (Turkey) [52], one in Africa (South Africa) [50], three in Latin-or South America (Mexico, Brazil, Peru, and Guatemala) [35, 36, 48], nine in Asia (India, Iran, China, Philippines, Thailand, Pakistan, and Malaysia) [40, 42–47, 49, 51] and one across a large number of countries [37]. Rubinstein et al. [36] conducted their study in Peru, Guatemala and Argentina, Lana et al. [35] in Spain and Mexico, and Ganesan et al., [37] included participants from 64 countries with 92 % from a developing country (mainly India). The number of participants ranged from 22 [50] to 69219 [37] with four trials enrolling less than 100 participants [46, 47, 50, 52]. In total 75930 people participated in all included studies. Fourteen studies enrolled adults over 18 years of age (range = 18 to 74 years), and one study recruited children and adolescents [35]. Study participants were healthy or at risk of developing diabetes or hypertension (*n* = 10 studies) [35–37, 40, 44, 46, 48, 49, 51, 52] or diabetic (*n* = 5 studies) [42, 43, 45, 47, 50]. Three studies enrolled only women [44, 50, 52] and one enrolled only men [40].

The e-& mHealth interventions were delivered via mobile phone text messages (*n* = 7 studies) [36, 40, 42, 43, 46, 49, 50], the Internet including websites and email (*n* = 6 studies) [35, 37, 44, 48, 51, 52], a website plus mobile phone text messages (*n* = 1) [35], mobile phone calls (*n* = 2) [45, 47], or mobile phone calls plus text messages (*n* = 1) [47]. In eight studies, limited face-to-face contact or printed media were also part of the e-& mHealth intervention [36, 40, 42, 43, 45, 46, 50, 51]. All studies provided information on the intensity of the e-& mHealth intervention. For example, in studies that used mobile phone text-messages as intervention delivery channel text-messaging frequency ranged from twice weekly to daily (mean = 4.5 text messages per week). Only five studies were theoretically framed [35, 36, 40, 44, 48] with the Transtheoretical Model of Behavior Change most frequently applied (*n* = 3 studies) [35, 36, 40]. However, in other interventions behaviour change techniques (BCTs) such as goal setting and self-monitoring were used to promote physical activity and healthy diets.

Both physical activity and diet outcomes were assessed in the majority of studies (*n* = 10) [35, 36, 40, 42, 43, 45, 47, 49, 51, 52] while four assessed only physical activity [37, 44, 46, 50] and one only diet [48]. Physical activity was assessed in various ways, however most studies used a self-report instrument including questionnaires, interviews and exercise diaries (*n* = 10) [35, 36, 40, 42, 43, 46, 47, 49, 51, 52]. The outcomes also varied greatly across studies: adherence to physical activity guidelines, time spent being active, physical activity-related energy expenditure, physical activity score, and exercise frequency. In one study step-count data were collected with a pedometer [50] while Sriramatr et al. [44] and Ganesan et al. [37] used both pedometer data and a questionnaire. Diet outcomes were only assessed subjectively via questionnaires, 24-h recall sheets and interviews. One study did not report how outcome data was assessed [45]. Secondary outcomes were assessed in ten studies [35–37, 40, 42, 43, 45, 46, 50, 51]. All these studies reported data on BMI and six studies also reported on waist circumference and/or body weight.

### Risk of bias within studies

The detailed CONSORT risk of bias assessment of the individual studies is presented in Additional file 2. On average the studies fulfilled 55.7 % of the assessment criteria (range = 28–88 %). Hence, overall the studies had a moderate risk of bias with just over half of the studies at high risk (*n* = 8 studies) [37, 42, 44, 45, 48, 50–52]. Few studies provided adequate information on intervention harms (*n* = 2) [40, 46], study protocol publication (*n* = 3) [35, 36, 51], study registration (*n* = 6) [35–37, 40, 46, 49], ancillary analyses (*n* = 5) [35–37, 40, 50], and randomization as well as blinding (*n* = 4) [36, 40, 43, 46].

### Intervention effectiveness

Of the 15 studies included, four reported no significant positive effects on either physical activity or diet outcomes following an e-& mHealth intervention [35, 42, 47, 50]. The majority of studies (*n* = 11) reported at least one significant positive effect on physical activity or diet outcomes (Table 2). No clear patterns emerged between those studies that were effective and those that were not. In terms of secondary outcomes BMI was measured in nine studies with two reporting a significant positive effect of an e-& mHealth intervention despite not focussing on weight loss [36, 51].

**Table 2:** CONSORT risk of bias assessment and effectiveness of e-&mHealth interventions on physical activity and diet outcomes

Study	Design	CONSORT score (percentage of fulfilled criteria)	e-& mHealth intervention effectiveness
Lana et al. 2014 [35]	3-group RCT	18 (72 %)	Diet: 0
			PA: 0
Rotheram-Borus et al. 2012 [50]	1-group pre-post-follow-up	7.5 (30 %)	Diet: Not included
			PA: 0
Ramachandran et al. 2013; Ram et al. 2014 [40, 41]	Prospective 2-group RCT	22 (88 %)	Diet: +
			PA: 0
Shetty et al. 2011 [42]	2-group RCT	7 (28 %)	Diet: 0
			PA: 0
Zolfaghari et al. 2012 [47]	2-group CT	13.5 (54 %)	Diet: 0
			PA: 0
Chen et al. 2014 [51]	1-group pre-post	10.5 (42 %)	Diet: +
			PA: +
Tamban et al. 2013 [43]	2-group RCT	15 (60 %)	Diet: +
			PA: +
Nurgul et al. 2015 [52]	1-group pre-post	8.5 (34 %)	Diet: +
			PA: +
Bombem et al. 2013 [48]	2-group CT	11.5 (46 %)	Diet: + (less decrease in diet quality)
			PA: Not included
Sriramatr et al. 2014 [44]	2-group RCT	12 (48 %)	Diet: Not included
			PA: +
Shahid et al. 2015 [45]	2-group RCT	11.5 (46 %)	Diet: +
			PA: +
Müller et al. 2016 [46]	2-group RCT	22 (88 %)	Diet: Not included
			PA: +
Rubinstein et al. 2016 [36]	2-group RCT	22 (88 %)	Diet: +
			PA: 0
Ganesan et al., 2016 [37]	1-group pre-post	12 (48 %)	Diet: Not included
			PA: +
Pfammatter et al., 2016 [49]	2-group CT	16 (64 %)	Diet: +
			PA: 0

*PA* physical activity, ‘+’ = significant positive effect of e-& mHealth intervention, ‘0’ = no significant effect of e-& mHealth intervention

## Discussion

Promoting healthy lifestyles is an effective public health strategy to address the NCD rise in developing countries [1]. Given the great proliferation of ICT in developing countries, the use of e-& mHealth approaches appear to be viable to promote physical activity and healthy diets [21–23]. The results of this systematic review suggest that e-& mHealth interventions can be effective in improving physical activity and diet quality in developing countries. Overall, this review showed that 50 % of the e-& mHealth interventions were effective in increasing physical activity, and 70 % of the identified interventions were effective in improving diet quality. This result is consistent with the findings from previous systematic reviews of e-and mHealth interventions conducted in developed countries [21, 24–26, 29, 31, 53–55]. The findings from this review also add to the overall evidence on the effectiveness of e-& mHealth interventions in developing countries that were conducted to assess other health outcomes (e.g., treatment adherence) [32, 56–61]. As the included studies used multiple intervention components, it was not possible to identify which specific components were associated with interventions effectiveness. However, most Internet-based interventions were effective in improving physical activity and/or diet while the evidence for mobile-phone interventions (text messages and counselling) was mixed.

The overall risk of bias of the included studies was moderate with just over half of the studies having a high risk of bias. Study quality or study bias are also a concern in studies conducted in developed countries. Most previous reviews on the effectiveness of e-& mHealth interventions to promote physical activity and healthy diets in developed countries reported that the included studies were of low to moderate quality or rather biased [28, 30, 55, 62, 63]. There was no association between the risk of bias score and the effectiveness of the e-& mHealth interventions. The four studies with the lowest risk of bias reported mixed results [35, 36, 40, 46] and the same is true for the studies with higher risk of bias.

Importantly, the majority of studies included in this review (*n* = 10) examined an intervention modality that is likely economically viable and has the potential to reach large numbers of people to address the steep increase of NCDs. While more high-quality RCTs are needed to broaden the evidence-base, it is also important to conduct real-life implementation studies, given that the majority of the included studies reported that the e-& mHealth interventions showed positive outcomes. The study by Ganesan et al. [37] provides an insightful example of real-life implementation of a low-cost e-& mHealth intervention to increase physical activity. The researchers included almost 70000 participants (92 % from developing countries) into their 100-day Stepathlon programme and found that daily step counts and weekly exercise participation increased greatly. This study was possible because academic researchers teamed up with the private sector and formed a strong collaborative network. To form academic-private partnerships in order to either conduct or upscale e-& mHealth interventions to increase physical activity and improve diet quality might also be an option for researchers in other developing countries. Benefits of such partnerships include sharing of expertise (academia: behavioural health knowledge, industry: intervention appeal and dissemination expertise) and data sharing which can lead to dynamic intervention development and adaptation [64]. Potential for such partnerships is especially high in countries where there is some preliminary evidence that such interventions are feasible. Feasibility information is available from Malaysia [65–67], Iraq [68], Pakistan [69] and South Africa [70] where the e-& mHealth interventions were well accepted and study participants found them useful.

A key strength of this review is that this is the first systematic review to investigate the effectiveness of e-& mHealth interventions to promote physical activity and healthy diets in developing countries. A further strength is that a large number of information sources were systematically searched to identify relevant studies and that the search was updated continuously (until August 2016). Additionally, data of the included studies were extracted in great detail despite the complex nature of some studies and large variations across studies.

A limitation of this review is that only articles published in English were included. Studies conducted in developing countries where English is not the first language might have been published in local languages and our search would not have identified them. The possibility of publication bias should also be acknowledged. As with all systematic reviews examining the efficacy of interventions, it is possible that some studies that did not find a beneficial effect of an e-& mHealth intervention have not been published [71].

Further, studies were only identified in a small number of countries and it is therefore difficult to generalize the review findings across developing countries (most studies were available from Asia). Additionally, most studies were conducted in upper-middle income countries (*n* = 10) and none in a low income country.

Due to the differences in study designs, outcome measures, lengths of studies, and study samples it was difficult to draw clear conclusions in this review. It is therefore important that researchers aim to examine the impact of e-& mHealth interventions to promote physical activity and healthy diets in a standardized manner. Applying the CONSORT guidelines that specifically highlight the importance of valid controls is strongly encouraged [38]. This will allow for a pooling of results to conduct a meta-analysis, and would reduce the risk of bias in the included studies.

Researchers should be encouraged to assess and report data on intervention acceptability and level of participation (as measured through web and app usage tracking). Only a limited number of studies included in this review presented this information. Knowing how well participants accept and are engaged in e-& mHealth interventions is essential because acceptability and user engagement are related to intervention effectiveness [72, 73]. Additionally, studies examining the cost-effectiveness and reach of e-& mHealth interventions in developing countries are currently lacking. These studies are needed to ensure that resource poor countries can impact health behaviours in a large number of people at an affordable cost. From this review, the impact of behavioural e-& mHealth interventions a) on clinical health outcomes and b) among patients versus non-patients is unclear and more research is warranted.

Finally, while behavioural e-& mHealth research in developed countries is well established [74], research in developing countries is only in its infancy. One indication for this is the small number of studies that were identified. In addition, the studies included in this review utilized mainly first generation technologies of the Internet and mobile phones to implement their interventions (*n* = 14). However, with the unprecedented expansion of mobile broadband in developing countries, e-& mHealth interventions with advanced technologies (e.g. smart phone apps and wearable activity trackers) [23] are likely to emerge [37]. One feasibility study conducted in South Africa already reported that a diet smartphone app for diabetic nephropathy patients is highly acceptable among dietitians [70]. It is important that researchers examine if these advanced technologies can be leveraged to promote physical activity and healthy diets in developing countries.

## Conclusions

In summary, using e-& mHealth approaches to promote physical activity and healthy diets in developing countries was effective in most studies included in this review. However, the interventions varied greatly in terms of geographic spread, intervention components evaluated, trial methods applied and study quality. Therefore, the findings from the included studies should be interpreted with caution, and more rigorous study designs are recommended for future e-& mHealth interventions.

## Additional files

Additional file 1: Cochrane Library Complete Search Strategy. (DOCX 18 kb)
Additional file 2: Risk of bias assessment using the CONSORT checklist. (DOCX 32 kb)

## Abbreviations

BMI: Body mass index
CONSORT: Consolidated Standards of Reporting Trials
CT: Controlled trial
e-& mHealth: Electronic and mobile health
ICT: Information and communication technology
MET: Metabolic Equivalent of task
NCD: Non-communicable disease
PRISMA: Preferred reporting items for systematic reviews and meta-analyses
RCT: Randomized controlled trial
