# Supplementary material for: The effectiveness of e-& mHealth interventions to promote physical activity and healthy diets in developing countries: A systematic review
Source: Int J Behav Nutr Phys Act. 2016 Oct 10;13:109. doi: 10.1186/s12966-016-0434-2 (PMC5057225; doi:10.1186/s12966-016-0434-2)
Supplement: Additional file 1: — Cochrane Library Complete Search Strategy. (DOCX 18 kb) [file 12966_2016_434_MOESM1_ESM.docx]

**Cochrane Library Complete Search Strategy**

Search (in title, abstract and keywords, 31.10.2015)

Limits: CENTRAL, HTA Database; 2000-2015; word variants enabled

| **Technology Terms (combined with OR)**  Technology Telemedicine Telehealth “information technology” “communication technology”  ICT “electronic health” eHealth Internet Online Tablet ipad Web “world wide web” Email Website “web based” “website delivered” “Mobile health” mHealth “Mobile phone” “short message service” SMS “multimedia messaging service” MMS “text message” App Smartphone “cell phone” “cellular phone” “picture message” tracker wearables | |
| --- | --- |
| **All Technology Terms combined with OR** | |
| technology or telemedicine or telehealth or "communication technology" or “information technology” or ICT or "electronic health" or eHealth or internet or online or tablet or ipad or web or "world wide web" or email or website or "web based" or "website delivered" or PDA or "mobile health" or mHealth or "mobile phone" or "short message service" or SMS or "multimedia messaging service" or MMS or "text message" or app or smartphone or "cell phone" or "cellular phone" or "picture message" or tracker or wearables:ti,ab,kw | 42690 |
| **PA and Diet Terms (combined with OR)**  “physical activity” “motor activity” “outdoor activity” Exercise “physical exercise” Walk  Sport “active transport” sit sedentary diet nutrition “healthy eating” “food intake” “Fruit and vegetable” “sugar-sweetened beverages” “sugar intake” “fat intake” “carbonated beverages”  Snack “Snack food” “food habit” “dietary habit” | |
| **All PA and Diet Terms combined with OR** | |
| "physical activity" or "motor activity" or “outdoor activity” or exercise or "physical exercise" or walk or sport or "active transport" or sit or sedentary or diet or nutrition or "health eating" or "food intake" or “fruit and vegetable” or "sugar-sweetened beverages" or “sugar intake” or “fat intake” or "carbonated beverages" or snack or "snack food" or "food habit" or “dietary habit”:ti,ab,kw | 71190 |
| **Developing country (combined with OR)**  “developing country” “developing world” “low-income country” “middle-income country”  “lower-middle income country” “upper-middle income country” “emerging country”  “resource limited” | |
| Afghanistan or Albania or Algeria or "American Samoa" or Andorra or Angola or "Antigua and Barbuda" or Armenia or Azerbaijan or Bangladesh or Belarus or Belize or Benin or Bhutan or Bolivia or "Bosnia and Herzegovina" or Botswana or Brazil or Bulgaria or "Burkina Faso" or Burundi or "Cabo Verde" or Cambodia or Cameroon or "Central African Republic" or Chad or China or Colombia or Comoros or Congo or "Costa Rica" or "Côte d'Ivoire" or Cuba or Djibouti or Dominica or "Dominican Republic" or Ecuador or Egypt or "El Salvador" or Eritrea or Ethiopia or Fiji or Gabon or Gambia or Georgia or Ghana or Grenada or Guatemala or Guinea or "Guinea-Bissau" or Guyana or Haiti or Honduras or India or Indonesia or Iran or Iraq or Jamaica or Jordan or Kazakhstan or Kenya or Kiribati or Korea or Kosovo or "Kyrgyz Republic" or Laos or Lebanon or Lesotho or Liberia or Libya or Macedonia or Madagascar or Malawi or Malaysia or Maldives or Mali or "Marshall Islands" or Mauritania or Mauritius or Mexico or Micronesia or Moldova or Mongolia or Montenegro or Morocco or Mozambique or Myanmar or Namibia or Nepal or Nicaragua or Niger or Nigeria or Pakistan or Palau or Panama or "Papua New Guinea" or Paraguay or Peru or Philippines or Romania or Rwanda or Samoa or "São Tomé and Principe" or Senegal or Serbia or "Sierra Leone" or "Solomon Islands" or Somalia or "South Africa" or "South Sudan" or "Sri Lanka" or "St. Lucia" or "St. Vincent and the Grenadines" or Sudan or Suriname or Swaziland or Syria or Tajikistan or Tanzania or Thailand or "Timor-Leste" or Togo or Tonga or Tunisia or Turkey or Turkmenistan or Tuvalu or Uganda or Ukraine or Uzbekistan or Vanuatu or Vietnam or Palestine or Yemen or Zambia or Zimbabwe:ti,ab,kw | 82994 |
| **All Developing Country Terms combined with OR** | |
| "developing country" or "developing world" or "low-income country" or "middle-income country" or "lower-middle income country" or "upper-middle income country" or "emerging country" or "resource limited" or Afghanistan or Albania or Algeria or "American Samoa" or Andorra or Angola or "Antigua and Barbuda" or Armenia or Azerbaijan or Bangladesh or Belarus or Belize or Benin or Bhutan or Bolivia or "Bosnia and Herzegovina" or Botswana or Brazil or Bulgaria or "Burkina Faso" or Burundi or "Cabo Verde" or Cambodia or Cameroon or "Central African Republic" or Chad or China or Colombia or Comoros or Congo or "Costa Rica" or "Côte d'Ivoire" or Cuba or Djibouti or Dominica or "Dominican Republic" or Ecuador or Egypt or "El Salvador" or Eritrea or Ethiopia or Fiji or Gabon or Gambia or Georgia or Ghana or Grenada or Guatemala or Guinea or "Guinea-Bissau" or Guyana or Haiti or Honduras or India or Indonesia or Iran or Iraq or Jamaica or Jordan or Kazakhstan or Kenya or Kiribati or Korea or Kosovo or "Kyrgyz Republic" or Laos or Lebanon or Lesotho or Liberia or Libya or Macedonia or Madagascar or Malawi or Malaysia or Maldives or Mali or "Marshall Islands" or Mauritania or Mauritius or Mexico or Micronesia or Moldova or Mongolia or Montenegro or Morocco or Mozambique or Myanmar or Namibia or Nepal or Nicaragua or Niger or Nigeria or Pakistan or Palau or Panama or "Papua New Guinea" or Paraguay or Peru or Philippines or Romania or Rwanda or Samoa or "São Tomé and Principe" or Senegal or Serbia or "Sierra Leone" or "Solomon Islands" or Somalia or "South Africa" or "South Sudan" or "Sri Lanka" or "St. Lucia" or "St. Vincent and the Grenadines" or Sudan or Suriname or Swaziland or Syria or Tajikistan or Tanzania or Thailand or "Timor-Leste" or Togo or Tonga or Tunisia or Turkey or Turkmenistan or Tuvalu or Uganda or Ukraine or Uzbekistan or Vanuatu or Vietnam or Palestine or Yemen or Zambia or Zimbabwe:ti,ab,kw | 84188 |
| **All Terms combined** | |
| #1 and #2 and #3 | 833 |
