# Supplementary material for: The effectiveness of e-& mHealth interventions to promote physical activity and healthy diets in developing countries: A systematic review
Source: Int J Behav Nutr Phys Act. 2016 Oct 10;13:109. doi: 10.1186/s12966-016-0434-2 (PMC5057225; doi:10.1186/s12966-016-0434-2)
Supplement: Additional file 2: — Risk of bias assessment using the CONSORT checklist. (DOCX 32 kb) [file 12966_2016_434_MOESM2_ESM.docx]

Risk of bias assessment using the CONSORT checklist

|  | Lana et al., 2014 | Rotheram-Borus et al., 2012 | Ramachandran et al., 2013; Ram et al., 2014 | Shetty et al., 2011 | Zolfaghari et al., 2012 | Chen et al., 2014 | Tamban et al., 2013 | Nurgul et al., 2015 | Bombem et al., 2013 | Sriramatr et al., 2014 | Shahid et al., 2015 | Müller et al., 2016 | Rubinstein et al., 2016 | Ganesan et al., 2016 | Pfammatter et al., 2016 |
| --- | --- | --- | --- | --- | --- | --- | --- | --- | --- | --- | --- | --- | --- | --- | --- |
| **Title and abstract** | + + | - + | + + | - + | - + | - + | + + | - + | - + | + + | + + | + + | + + | - + | - + |
| a) identification as randomized trial in title; b) structured summary |  |  |  |  |  |  |  |  |  |  |  |  |  |  |  |
| **Introduction** | + + | + + | + + | ++ | + + | + - | + + | + - | + + | + + | + + | + + | + + | + + | + + |
| a) scientific background/rationale; b) specific objectives/hypotheses |  |  |  |  |  |  |  |  |  |  |  |  |  |  |  |
| **Methods – *trial design*** | + - | - - | + - | - - | + - | + - | + - | + - | + - | + - | + - | + - | + - | + - | + - |
| a) description of trial design; b) changes in methods after trial commencement |  |  |  |  |  |  |  |  |  |  |  |  |  |  |  |
| ***Participants*** | - + | - - | + + | + - | + + | - + | + - | + + | + + | + - | + + | + + | + + | + + | + + |
| a) eligibility criteria; b) settings and locations of data collection |  |  |  |  |  |  |  |  |  |  |  |  |  |  |  |
| ***Interventions*** | + | + | + | - | + | + | - | - | + | + | + | + | + | + | + |
| Descriptions of sufficient details to allow replication |  |  |  |  |  |  |  |  |  |  |  |  |  |  |  |
| ***Outcomes*** | + + | + - | + - | + - | + - | + - | + - | + - | + - | + - | + - | + - | + - | + - | + - |
| a) pre-specified primary and secondary outcomes; b) changes to outcomes after trial commencement |  |  |  |  |  |  |  |  |  |  |  |  |  |  |  |
| ***Sample size*** | + | - | + + | - |  | - - | + |  | + | + | + | + | + + | - - | + |
| a) how sample size was determined; b) if applicable, interim analysis/ stopping guidelines |  |  |  |  |  |  |  |  |  |  |  |  |  |  |  |
| **Randomization – *sequence generation*** | + + | - - | + + | + - | + + | - - | + + | - - | - - | + - | - - | + + | + + | - - | - - |
| a) method used; b) type of randomization including any type of restriction |  |  |  |  |  |  |  |  |  |  |  |  |  |  |  |
| ***Allocation concealment mechanism*** | - | - | + | - | - | - | + | - - | - | - | - - | + | + | - | - |
| Implementation of random allocation sequence, including concealment |  |  |  |  |  |  |  |  |  |  |  |  |  |  |  |
| ***Implementation*** | - | - | + | - | - | - | + | - - | - | - | - | + | + | - | - |
| Who generated random allocation sequence, who enrolled participants, who assigned participants |  |  |  |  |  |  |  |  |  |  |  |  |  |  |  |
| **Blinding** | - | - | + | - | - - | - - | + + | - - | - - | - - | - - | + | + | - - | + |
| a) if done, who was blinded and how; b) if relevant, similarity of interventions |  |  |  |  |  |  |  |  |  |  |  |  |  |  |  |
| **Statistical methods** | + + | + - | + + | + - | + - | + + | + - | + - | + - | + - | + + | + + | + + | + + | + + |
| Statistical methods used a) for primary outcomes; b) additional analyses |  |  |  |  |  |  |  |  |  |  |  |  |  |  |  |
| **Results – *participant flow*** | + - | + + | + + | + - | + + | + - | + + | + - | + - | + + | - - | + + | + + | + - | + - |
| a) number of participants randomized, receiving treatment, and analyzed; b) losses and exclusions, with reasons |  |  |  |  |  |  |  |  |  |  |  |  |  |  |  |
| ***Recruitment*** | + - | - - | + - | - - | + - | + - | - - | + - | + - | - - | + - | + + | + + | - - | + + |
| a) dates of recruitment and follow-up; b) why the trial ended |  |  |  |  |  |  |  |  |  |  |  |  |  |  |  |
| ***Baseline data*** | + | + | + | - | + | - | + | + | + | - | + | + | + | + | + + |
| A table with baseline demographic and clinical characteristics for each group |  |  |  |  |  |  |  |  |  |  |  |  |  |  |  |
| ***Numbers analyzed*** | + | + | + | + | + | + | + | + | + | + | + | + | + | - | + |
| For each group, number of participants included in each analyses |  |  |  |  |  |  |  |  |  |  |  |  |  |  |  |
| ***Outcomes and estimation*** | - - | - | + - | - - | - - | - - | - - | - - | + - | + - | + | + | + | + | + |
| a) results for each group, and the estimated effect size and its precision; b) absolute and relative effect sizes for binary outcomes |  |  |  |  |  |  |  |  |  |  |  |  |  |  |  |
| ***Ancillary analyses*** | + | + | + | - | - | - | - | - | - | - | - | - | + | + | - |
| Results of any other analyses performed, distinguishing pre-specified from exploratory |  |  |  |  |  |  |  |  |  |  |  |  |  |  |  |
| ***Harms*** | - | - | + | - | - | - | - | - | - | - | - | + | - | - | - |
| Harms or unintended effects in each group |  |  |  |  |  |  |  |  |  |  |  |  |  |  |  |
| **Discussion - *Limitations*** | + | - | + | + | + | + | + | - | + | + | + | + | + | + | + |
| Trial limitations/ bias/ multiplicity of analyses |  |  |  |  |  |  |  |  |  |  |  |  |  |  |  |
| ***Generalisability*** | + | - | + | - | + | + | - | + | - | - | - | + | + | - | + |
| Generalisability (external validity/ applicability) of findings |  |  |  |  |  |  |  |  |  |  |  |  |  |  |  |
| ***Interpretation*** | + | - | + | + | + | + | + | + | - | + | - | + | + | + | + |
| Consistent with results and balanced |  |  |  |  |  |  |  |  |  |  |  |  |  |  |  |
| **Other information - *Registration*** | + | - | + | - | - | - | - | - | - | - | - | + | + | + | + |
| Registration number and name of registry |  |  |  |  |  |  |  |  |  |  |  |  |  |  |  |
| ***Protocol*** | + | - | - | - | - | + | - | - | - | - | - | - | + | - | - |
| Where full trial protocol can be accessed |  |  |  |  |  |  |  |  |  |  |  |  |  |  |  |
| ***Funding*** | + | - | + | - | + | - | + | + | + | + | - | + | - | - | + |
| Sources of funding/ role of funders |  |  |  |  |  |  |  |  |  |  |  |  |  |  |  |
| Number of criteria satisfied (in percent) | 18 (72%) | 7.5  (30%) | 22  (88%) | 7 (28%) | 13.5 (54%) | 10.5 (42%) | 15 (60%) | 8.5 (34%) | 11.5 (46%) | 12  (48%) | 11.5 (46%) | 22 (88%) | 22  (88%) | 12  (48%) | 16  (64%) |
